# Energy–Tissue Interaction in Physiotherapy: A Proposed Conceptual Framework for Electrophysical Agents

**DOI:** 10.3390/jcm15218216

**Published:** 2026-10-22

**Authors:** Panagiotis Rentzias, Stelios Hadjisavvas, Kyriakos Pavlou, Charalambos Papacharalambous, George M. Pamboris, Christos Karagiannis

**Affiliations:** 1 Department of Health Sciences, School of Life and Health Sciences, European University Cyprus, 6 Diogenous Str., 2404 Nicosia, Cyprus; k.pavlou@external.euc.ac.cy (K.P.); c.papacharalambous@euc.ac.cy (C.P.); g.pamboris@euc.ac.cy (G.M.P.); c.karayiannis@euc.ac.cy (C.K.); 2 Department of Life and Health Sciences, School of Health Sciences, Frederick University, 3080 Limassol, Cyprus; hsc.hs@frederick.ac.cy

**Keywords:** electrophysical agents, energy–tissue interaction, physiotherapy, rehabilitation, biophysical mechanisms

## Abstract

Electrophysical agents (EPAs) are widely used in rehabilitation, yet their selection is frequently guided by diagnostic labels, established protocols, or device-specific parameters, with comparatively limited consideration of how the delivered energy physically interacts with the intended target tissue. This narrative review examines energy–tissue interactions underlying six commonly used EPAs: therapeutic ultrasound, photobiomodulation (LASER), diathermy, capacitive–resistive electric transfer (TECAR/CRET), extracorporeal shockwave therapy (ESWT), and pulsed electromagnetic fields (PEMF). Relevant literature was identified through searches of PubMed and Scopus, prioritising publications from 2016 to 2026 while incorporating earlier foundational studies. Experimental investigations, computational models, and relevant reviews were narratively synthesised to examine energy propagation, attenuation, distribution, tissue-specific interactions, and the resulting physical and biological stimuli. Based on this synthesis, the Tissue Energy Interaction Framework (TEIF) is introduced as a proposed conceptual model requiring further validation rather than an established clinical decision-making tool. The framework describes a pathway from energy generation and delivery through tissue propagation, attenuation/distribution, target interaction, physical or biological stimulus, biological response, and potential clinical effect. The reviewed evidence indicates that tissue depth, composition, interfaces, and modality-specific characteristics substantially influence the physical stimulus reaching a therapeutic target. Energy penetration should therefore be distinguished from effective target interaction, while measurable interaction does not necessarily establish clinical effectiveness. The TEIF proposes that physical and biological plausibility should inform EPA selection alongside, but never supersede, evidence of clinical effectiveness and patient-specific considerations. Further research is required to validate the framework and determine whether tissue-informed EPA selection improves clinical outcomes.

## 1. Introduction

Over the past few decades, numerous studies have investigated the use of electrophysical agents (EPAs) in the management of musculoskeletal disorders [1,2]. EPAs encompass a range of therapeutic modalities that deliver different forms of physical energy and may be employed by physiotherapists and other healthcare professionals as components of rehabilitation programmes [3]. The International Society for Electrophysical Agents in Physical Therapy (ISEAP), associated with the World Confederation for Physical Therapy has contributed to the professional recognition and development of electrophysical agents within physiotherapy practice [4]. Nevertheless, their clinical application should be guided by evidence of effectiveness, patient-specific considerations, and an understanding of the physical mechanisms through which the delivered energy interacts with biological tissues [5,6].

The clinical application of EPAs involves two interrelated considerations. The first concerns the available evidence supporting their effectiveness for specific clinical conditions, while the second concerns the biophysical mechanisms governing energy delivery, propagation, and interaction with the intended therapeutic target [7,8]. These considerations are complementary rather than competing. Although clinical research has examined the effectiveness of various EPAs, the extent to which tissue-specific energy interactions are incorporated into treatment selection and parameter prescription remains an important area for further investigation.

The role of EPAs in contemporary physiotherapy continues to be debated, particularly regarding the strength of evidence supporting individual modalities and their appropriate integration into multimodal rehabilitation programmes [6,9,10]. Differences in clinical practice may reflect variations in professional training, familiarity with emerging evidence, equipment availability, healthcare resources, and treatment preferences. Importantly, the evidence supporting EPAs varies according to the modality, clinical condition, treatment parameters, and outcomes assessed. Consequently, some clinical practice guidelines recommend particular EPAs for selected indications, whereas others do not support their routine use because of insufficient or inconsistent evidence [11]. These differences highlight the importance of considering both clinical effectiveness and the physical rationale underlying treatment application.

### The “Standard Treatment/Protocol” Syndrome

Over the past several decades, the composition of what is commonly referred to as standard or conservative physiotherapy has continuously evolved. Interventions that were once considered innovative therapeutic approaches have gradually become incorporated into routine clinical practice and may subsequently be included as components of standard care. Therapeutic ultrasound provides a representative example. Initially introduced as a primary treatment modality for musculoskeletal disorders, ultrasound has been extensively investigated over several decades and continues to appear within conventional physiotherapy protocols, despite ongoing debate regarding its clinical effectiveness for particular conditions [12,13].

A similar evolution can be observed across many electrophysical agents and rehabilitation interventions. Contemporary randomized controlled trials frequently describe standard or conservative treatment as a combination of therapeutic exercise, manual therapy, superficial heat, electrotherapy, and other physical modalities [14]. Manual therapy and structured exercise are also frequently incorporated into evidence-informed musculoskeletal rehabilitation [2,15], while technologies such as extracorporeal shock wave therapy (ESWT) have become established treatment options for selected clinical indications [16].

Consequently, the composition of standard care should be regarded as dynamic rather than fixed. As new interventions are introduced, they may be incorporated into existing rehabilitation programmes, resulting in multimodal treatment approaches. However, inclusion within a conventional treatment protocol does not necessarily establish the effectiveness or biophysical suitability of every component for a particular clinical presentation. The rationale for selecting individual interventions should therefore remain open to reassessment as scientific knowledge and clinical evidence evolve. Therefore, many electrophysical agents—including ultrasound, TENS, and various forms of diathermy—remain embedded within contemporary physiotherapy practice, even though they are no longer presented as stand-alone primary interventions.

To describe the potential tendency for therapeutic modalities to become routinely incorporated into clinical protocols without periodic reassessment of their underlying rationale, the authors propose the term “Standard Treatment/Protocol Syndrome.” This is an author-proposed conceptual description rather than an established or empirically validated clinical construct. It does not imply that standard physiotherapy protocols are inherently inappropriate or that clinicians routinely disregard scientific evidence. Rather, it highlights the possibility that familiarity with established treatment combinations may sometimes reduce attention to the specific justification for including an individual modality. In the case of EPAs, such reassessment should consider not only clinical effectiveness but also the physical principles governing energy delivery and interaction with biological tissues.

This perspective raises an important question concerning EPA selection. Beyond determining whether a modality is conventionally included within a treatment protocol, clinicians should consider whether the physical characteristics of the delivered energy are compatible with the intended anatomical target and whether sufficient clinical evidence supports its application. Tissue depth, composition, anatomical interfaces, and modality-specific treatment parameters may influence the physical stimulus reaching the target. Understanding these interactions may therefore complement evidence-based clinical decision-making by providing a more explicit biophysical rationale for treatment selection.

The aim of this narrative review is to examine the physical principles governing energy–tissue interactions across six commonly used electrophysical agents and to introduce the Tissue Energy Interaction Framework (TEIF) as a proposed conceptual model for understanding the relationship between energy delivery, tissue-specific interaction, biological response, and potential clinical effects. By distinguishing physical plausibility from demonstrated clinical effectiveness, the framework seeks to support more transparent, target-centred reasoning in EPA prescription. The TEIF is not presented as a validated clinical decision-making tool, and its potential clinical utility requires further experimental and clinical investigation.

## 2. Materials and Methods

### 2.1. Review Design and Conceptual Approach

This study was designed as a structured narrative review with a conceptual synthesis of the physical, biological, and clinical evidence concerning electrophysical agents (EPAs) used in physiotherapy. The review examined six modalities: therapeutic ultrasound, photobiomodulation (PBM), shortwave and microwave diathermy, capacitive–resistive electric transfer therapy (TECAR), extracorporeal shock wave therapy (ESWT), and pulsed electromagnetic field therapy (PEMF).

The primary objective was to examine how the physical characteristics of externally delivered energy interact with the properties of biological tissues and how these interactions may contribute to subsequent biological and clinical effects. Attention was given to energy generation, delivery, propagation, attenuation, distribution, and interaction with target tissues.

Because the modalities differ substantially in their underlying physical mechanisms, a narrative synthesis was considered more appropriate than quantitative pooling. The review integrated experimental, computational, physiological, and clinical evidence while distinguishing directly measured physical interactions from inferred biological mechanisms and patient-relevant therapeutic outcomes.

The Tissue Energy Interaction Framework (TEIF) was developed as an interpretative conceptual model to organize these relationships. The framework is a hypothesis-generating synthesis and has not undergone independent empirical validation.

### 2.2. Literature Search Strategy

A structured literature search was conducted using PubMed/MEDLINE and Scopus to identify publications relevant to the physical and biological interactions of the six electrophysical modalities.

The searches were organized separately by modality to accommodate differences in terminology, physical principles, and experimental methods. Search concepts combined modality-specific terms with terminology relating to tissue interactions, energy transmission, absorption, attenuation, penetration, thermal or mechanical effects, electromagnetic properties, and biological responses, as applicable.

Modality-specific search strategies combined terms identifying each electrophysical agent with terms describing energy–tissue interactions. Boolean operators (AND, OR) were used to combine modality-related terms and interaction-related terms. PubMed searches incorporated Title/Abstract fields and relevant Medical Subject Headings (MeSH), where applicable, while Scopus searches used the TITLE-ABS-KEY field. The searches addressed energy propagation, attenuation, absorption, optical scattering, tissue heating, electrical field distribution, mechanical stimulation, and biological responses. Full database-specific search strategies are provided in Supplementary Table S1. Although the Scopus search employed publication-year filters covering 2016–2026, the predefined eligibility cutoff of 1 September 2026 was applied during study selection, and publications appearing after this date were excluded from the final evidence synthesis.

The database searches were organised by electrophysical modality using combinations of modality-specific terms and concepts relating to energy–tissue interactions. Where complete original database search histories were unavailable, the search strategies presented in Supplementary Table S1 were retrospectively reconstructed from the documented search terminology and Boolean structure. These reconstructed strategies are explicitly identified and should be distinguished from the original executed searches. Accordingly, the recorded retrieval counts represent the initial literature searches and have not been independently verified as the results of the reconstructed queries. The reconstructed strategies are provided to improve methodological transparency and facilitate replication of the search approach, while acknowledging this limitation in exact reproducibility.

The initial searches yielded 3995 database records, comprising 1029 from PubMed and 2966 from Scopus. After deduplication using digital object identifiers (DOIs) and normalized article titles, 3271 unique records remained.

The distribution of retrieved records by modality and database is presented in Table 1.

### 2.3. Eligibility Criteria and Study Selection

Publications were considered potentially relevant when they addressed at least one of the following domains:

1. Physical characteristics of energy delivery, including frequency, wavelength, pulse characteristics, intensity, power, energy density, or treatment configuration.
2. Energy propagation, attenuation, absorption, reflection, scattering, or distribution in biological tissues or relevant experimental models.
3. Tissue-dependent interactions associated with anatomical composition, conductivity, acoustic impedance, optical properties, or other relevant physical characteristics.
4. Experimentally observed thermal, mechanical, electrical, photochemical, or cellular responses following exposure.
5. Clinical outcomes that could inform interpretation of the relationship between physical exposure, biological response, and therapeutic effectiveness.

Eligible evidence types included original experimental investigations, human physiological studies, animal and ex vivo studies, computational simulations, and relevant clinical investigations. Systematic reviews and narrative reviews were considered for contextual interpretation and identification of supporting primary literature.

Studies without a meaningful relationship to the review objectives, publications outside the predefined date range, and reports lacking sufficient information to interpret the relevant exposure or interaction were considered for exclusion.

Study selection was organized by modality. Titles and abstracts were used to identify potentially relevant publications, followed by full-text examination of selected articles. Final eligibility decisions were based on the relevance of each publication to the physical, biological, or clinical components of the proposed framework.

### 2.4. Data Extraction and Evidence Organization

Evidence was organized using a structured extraction matrix developed for the purposes of this review. The matrix was intended to capture study characteristics, experimental or clinical models, exposure parameters, anatomical targets, investigated tissue properties, measured outcomes, principal findings, and methodological limitations.

Where available, information was extracted on the characteristics of the applied energy, including frequency, wavelength, intensity, power, energy flux density, pulse duration, treatment duration, applicator configuration, and electrode placement.

Outcome measures were classified into two distinct domains:

- Physical interaction: measurements or estimates of energy distribution, acoustic pressure, electric fields, optical penetration, temperature changes, mechanical stress, or related physical quantities.
- Biological response: cellular signalling, tissue perfusion, inflammatory mediators, extracellular matrix changes, and other physiological or biochemical effects.

These domains were considered separately to avoid interpreting energy delivery or experimentally observed biological activity as direct evidence of clinical effectiveness.

### 2.5. Development of the Tissue Energy Interaction Framework

The TEIF was constructed by integrating established physical principles with findings from the retrieved literature. The framework describes a sequence of interconnected stages: energy generation, delivery to the body, propagation through biological tissues, attenuation and spatial distribution, interaction with target structures, conversion into physical or biochemical stimuli, biological response, and potential clinical effect.

For each modality, the synthesis examined how treatment parameters and tissue characteristics could modify these stages.

The framework was intended to identify plausible mechanisms, and evidence gaps rather than to establish deterministic relationships between energy exposure and treatment outcomes. Accordingly, the presence of a measurable physical interaction was not assumed to indicate a beneficial biological response, and a biological response was not assumed to establish clinical efficacy.

### 2.6. Comparative Tissue–Modality Analysis

A comparative matrix was developed to summarize the relationships between electrophysical modalities and selected biological tissues.

The assessment considered the physical characteristics of each modality, tissue-dependent mechanisms of interaction, and the availability of direct experimental or computational evidence. Evidence obtained from human participants, cadaveric specimens, isolated tissues, in vitro systems, and numerical simulations was interpreted according to its respective methodological limitations.

Where qualitative interaction descriptors were used, they represented author-derived interpretations of the available evidence rather than validated measurements or standardized effect-size categories. Such descriptors were not considered interchangeable across modalities because acoustic, optical, radiofrequency, mechanical, and electromagnetic exposures are characterized by different physical quantities.

Insufficient or indirect evidence was identified as uncertain rather than interpreted as demonstrating the absence of interaction.

### 2.7. Evidence Synthesis and Methodological Considerations

A narrative synthesis was undertaken because substantial heterogeneity was anticipated across modalities, study designs, exposure protocols, tissue models, and outcome measures.

Evidence was interpreted according to its directness, experimental context, measurement methods, and applicability to living human tissues. Particular caution was applied when extrapolating from computational simulations, in vitro experiments, or cadaveric models to clinical conditions.

The synthesis also considered whether studies measured the physical interaction directly or inferred it from secondary physiological or clinical outcomes.

No quantitative meta-analysis was planned. The review did not apply a validated quantitative scoring system to rank tissue-specific energy interactions, and the proposed TEIF was not subjected to prospective validation.

## 3. Energy–Tissue Interaction

The therapeutic application of electrophysical agents (EPAs) involves the delivery of physical energy to biological tissues, where its propagation and subsequent interactions depend on both the characteristics of the energy source and the properties of the exposed medium. Following application, energy may undergo transmission, reflection, refraction, scattering, attenuation, or absorption, depending on its physical form and the tissues encountered [17]. Consequently, the energy emitted by a therapeutic device does not necessarily correspond to the energy available at the intended anatomical target.

The mechanisms through which physical energy interacts with biological tissues differ among electrophysical modalities. Optical energy undergoes wavelength-dependent absorption and scattering [18], whereas radiofrequency and electromagnetic exposures are influenced by electrical and dielectric properties and may produce thermal effects under appropriate exposure conditions [19]. Acoustic modalities can produce thermal and mechanical effects, including those associated with acoustic radiation force, streaming, and cavitation [20]. These processes illustrate that energy delivery represents only the initial stage of a more complex sequence of physical and biological events.

Within this context, three distinct levels should be considered: the physical availability and interaction of energy at the target, the subsequent biological response, and the potential clinical effect. Demonstrating that energy reaches a particular anatomical structure does not establish that a therapeutically relevant physical stimulus has been produced, while evidence of a biological response does not independently establish clinical effectiveness. Understanding this sequence provides a conceptual basis for selecting electrophysical modalities according to the intended target and the physical interaction required, rather than relying exclusively on device output or standardized treatment protocols [21].

## 4. Tissue Characteristics

The physical properties of biological tissues are important determinants of energy propagation and interaction. Differences in water, lipid, protein, and mineral content, together with variations in tissue density, structural organization, and anatomical interfaces, contribute to tissue-dependent acoustic, optical, and electrical behaviour [22]. These properties are considered separately below because their relative importance differs according to the form of energy applied.

Optical properties: The interaction of light with biological tissues is primarily governed by wavelength-dependent absorption and scattering. Endogenous chromophores, including haemoglobin, melanin, and water, contribute to optical absorption, while lipids and other tissue constituents may also influence absorption within particular spectral regions [23,24]. Scattering is influenced by refractive-index variations and tissue microstructure, including the organization of cells and extracellular components [24]. Consequently, the spatial distribution of optical energy depends on both wavelength and the optical characteristics of the tissues traversed.

Electrical and dielectric properties: Biological tissues differ in electrical conductivity and permittivity, and these properties are frequency dependent [22]. Ionic content, water distribution, cellular organization, and membrane characteristics contribute to the electrical behaviour of tissues. Experimental investigations have further demonstrated the importance of frequency-dependent dielectric characterization and accurate measurement techniques when evaluating the electrical behaviour of biological tissues [23]. For example, muscle and adipose tissue may exhibit substantially different conductivity characteristics, while muscle conductivity may also vary with fibre orientation. During radiofrequency exposure, these properties influence electric-field distribution, current pathways, and local energy dissipation. However, the resulting spatial pattern also depends on applicator geometry, electrode configuration, and exposure frequency. Therefore, tissue conductivity alone should not be interpreted as a direct measure of local heating or therapeutic effectiveness.

Acoustic properties: Acoustic-wave propagation is influenced by tissue density, sound speed, acoustic impedance, and attenuation. At interfaces between tissues with different acoustic impedances, part of the incident wave may be reflected, with the reflected proportion depending on the magnitude of the impedance mismatch and the angle of incidence. Within tissues, acoustic attenuation may result from absorption and scattering, while the distribution of mechanical stress depends on the incident acoustic field and local tissue characteristics [20]. These interactions are particularly relevant to therapeutic ultrasound and extracorporeal shock wave therapy, although the two modalities differ considerably in their acoustic exposure characteristics.

Taken together, these tissue-specific properties provide the physical basis for understanding why a given electrophysical modality may produce different exposure distributions across skin, adipose tissue, muscle, tendon, and bone. Their interpretation requires consideration of the particular form of energy applied and the anatomical structures encountered, rather than assuming uniform tissue responses across modalities.

## 5. Energy Characteristics

Electrophysical agents deliver energy in fundamentally different physical forms, and the characteristics of this energy determine its propagation, spatial distribution and potential biological effects. The modalities considered in the present framework can broadly be classified as acoustic/mechanical energy (therapeutic ultrasound and extracorporeal shock wave therapy), photonic energy (LASER/photobiomodulation), electromagnetic or radiofrequency energy (diathermy and TECAR), and magnetic-field-based energy (pulsed electromagnetic field therapy). Although these modalities are collectively classified as electrophysical agents, their energetic characteristics differ substantially. Consequently, comparison between modalities based solely on device output or applied dose may overlook important differences in the physical nature of the delivered energy [25,26,27].

Therapeutic ultrasound delivers acoustic energy through propagating pressure waves. The acoustic field is characterised by parameters including frequency, acoustic pressure, intensity, pulse duration, pulse repetition frequency, duty cycle and exposure duration. These parameters determine both the spatial and temporal characteristics of ultrasound exposure and influence its potential thermal and mechanical effects [25,28]. Absorption of acoustic energy may result in heating, whereas mechanical effects can arise through mechanisms including acoustic radiation force, streaming and cavitation. Therefore, therapeutic ultrasound cannot be adequately characterised by a single parameter such as intensity or frequency; rather, its physical stimulus is determined by the combination of multiple exposure characteristics.

Extracorporeal shock wave therapy (ESWT) also involves the propagation of acoustic/mechanical energy, but its physical characteristics differ substantially from conventional therapeutic ultrasound. Shock waves are characterised by short-duration pressure pulses with rapid pressure changes and substantial pressure amplitudes. Rather than producing a continuous or periodically oscillating acoustic field comparable to therapeutic ultrasound, shock waves transfer mechanical momentum during brief acoustic events. The resulting mechanical forces depend partly on momentum transfer at acoustic interfaces, providing a physical basis through which shock-wave energy may subsequently initiate mechanotransduction processes [29]. Thus, therapeutic ultrasound and ESWT should not be considered interchangeable merely because both involve acoustic energy.

LASER and photobiomodulation therapy deliver electromagnetic energy in the form of photons, commonly within the red and near-infrared regions of the electromagnetic spectrum. The energetic characteristics of light are strongly dependent on wavelength, while clinical exposure is additionally determined by parameters such as optical power, irradiance, radiant exposure, exposure duration, beam area and mode of delivery. Photobiological responses require photons to reach and interact with appropriate molecular photoacceptors; therefore, both wavelength and delivered optical dose are important determinants of the resulting stimulus [27]. Importantly, the optical energy emitted by the device should be distinguished from the energy ultimately available for interaction within the biological target.

Diathermy employs high-frequency electromagnetic energy to generate electrical and thermal effects within biological tissues. In shortwave diathermy, energy delivery may be continuous or pulsed and can be applied through capacitive or inductive techniques. Consequently, frequency alone is insufficient to describe the energetic stimulus. Mode of application, power, pulse characteristics, electrode configuration and treatment duration can substantially modify the resulting energy distribution. Evidence from thermal studies demonstrates that different shortwave diathermy techniques and settings produce markedly different temperature responses, emphasising the importance of treatment parameters in determining the physical stimulus generated by the modality [30].

TECAR, commonly described as capacitive–resistive electric transfer, similarly applies radiofrequency electrical energy but uses an active electrode and a return electrode to establish alternating current flow through biological tissues. Its energetic characteristics are influenced by frequency, applied power, electrode configuration, treatment duration and the capacitive or resistive mode of application. Experimental studies have demonstrated that changes in applied power can modify both electric current transmission and temperature responses in superficial and deeper structures [31]. These findings further illustrate that the nominal frequency of an electrophysical agent alone does not adequately describe the energy ultimately delivered during treatment.

Magnetic therapy, particularly pulsed electromagnetic field therapy (PEMF), differs from modalities that primarily rely on direct acoustic, optical or electrical energy transfer. Time-varying magnetic fields can penetrate biological tissues and induce secondary electric fields and currents through electromagnetic induction. The characteristics of the resulting stimulus depend on several parameters, including magnetic flux density, frequency, waveform, pulse duration, rate of change in the magnetic field and coil geometry [26]. Accordingly, magnetic field strength alone provides an incomplete description of PEMF exposure, and considerable variability in these parameters across devices and experimental protocols may contribute to differences in observed biological responses.

Collectively, these modalities demonstrate that the term electrophysical agent encompasses distinctly different forms of physical energy. Frequency, wavelength, intensity, pressure, power, pulse characteristics, waveform, exposure duration and applicator configuration collectively determine the physical stimulus generated by each modality. Therefore, the energy emitted by an electrophysical device represents only the starting point of the therapeutic process. Its clinical relevance ultimately depends on how these energy characteristics interact with the physical properties of the tissues encountered during propagation and at the intended therapeutic target.

## 6. Modality-Specific Energy–Tissue Interactions

The application of physical energy to biological tissues represents a dynamic process in which the characteristics of the energy source, the pathway of propagation and the physical properties of the exposed tissues collectively determine the resulting stimulus. Consequently, the energy produced by an electrophysical device should not be considered equivalent to the energy ultimately delivered to, or interacting with, the intended therapeutic target. Modern approaches to non-ionising energy dosimetry increasingly recognise that meaningful characterisation of an exposure requires consideration of the internal physical quantities generated within tissues rather than reliance solely on the nominal output of the device [32].

Following application, physical energy undergoes a sequence of interactions determined by both its physical form and the characteristics of the biological medium. Electromagnetic energy may be absorbed and converted into heat, optical energy may undergo absorption and scattering, and acoustic energy may generate thermal and mechanical effects through processes including absorption, radiation force and cavitation [33,34]. These interactions are not spatially uniform because the human body consists of heterogeneous tissues and interfaces. Consequently, the distribution of energy within the body may differ substantially from the distribution expected solely from the energy emitted at the applicator.

A distinction should therefore be made between energy delivery, penetration and target interaction. Penetration describes the ability of an energy form to propagate through biological tissues, whereas target interaction describes the physical process occurring when that energy reaches and couples with a specific tissue or cellular structure. An energy form may penetrate relatively deeply while only a proportion of the initial energy interacts with the intended target. Conversely, substantial local interaction may occur where physical properties change between adjacent tissues or where particular tissue constituents preferentially interact with the delivered energy. The biological response therefore depends not simply on whether energy reaches a particular anatomical depth, but on the magnitude, spatial distribution and mechanism of the physical interaction occurring at that location [32].

Importantly, different forms of physical energy cannot be evaluated using a single universal model of tissue interaction. Electromagnetic, optical, acoustic and mechanical energies differ in their propagation mechanisms and in the processes through which they transfer energy or generate physical forces within biological tissues. Energy-based therapeutic research demonstrates that different physical sources can produce distinct patterns of tissue interaction and energy deposition despite ultimately producing apparently similar biological outcomes, such as thermal or mechanical responses [35]. Therefore, the concept of “absorption” alone is insufficient to describe all electrophysical agents; the broader concept of energy–tissue interaction incorporates absorption, scattering, reflection, energy conversion, induced electrical effects and mechanical energy transfer.

From a physiotherapy perspective, these principles support a shift from modality-centred prescription toward target-centred energy selection. Rather than asking which electrophysical agent is conventionally recommended for a particular diagnosis, the clinician should first identify the anatomical and biological target and then consider whether the physical characteristics of the selected energy permit a meaningful interaction with that target. This requires consideration not only of target depth, but also of the tissues located between the applicator and target, the interfaces encountered during propagation and the mechanism through which the delivered energy is expected to generate a biological response. Energy–tissue compatibility should therefore be regarded as a component of the biophysical rationale underlying electrophysical-agent selection, while recognising that physical compatibility alone does not establish clinical effectiveness. The following sections examine this relationship for individual electrophysical agents and explore how differences in tissue composition and energy characteristics may influence their suitability for specific therapeutic targets.

### 6.1. Therapeutic Ultrasound–Tissue Interaction

Therapeutic ultrasound introduces acoustic energy into biological tissues through propagating pressure waves. Once the acoustic wave enters the body, its energy is progressively modified by transmission, reflection, scattering and absorption. The magnitude of these processes depends on both the characteristics of the ultrasound field and the acoustic properties of the tissues encountered. Frequency is particularly important because attenuation generally increases with increasing frequency; consequently, higher-frequency ultrasound deposits a greater proportion of its energy superficially, whereas lower-frequency ultrasound preserves a greater proportion of the incident energy during propagation toward deeper structures [34].

Importantly, attenuation is strongly tissue dependent. At 1 MHz, reported attenuation coefficients are approximately 0.63 dB/cm for adipose tissue, 0.96–1.40 dB/cm for skeletal muscle, depending on fibre orientation, 1.57 dB/cm for connective tissue and approximately 15 dB/cm for bone. Absorption represents a substantial component of this attenuation and results in conversion of acoustic energy into heat. Therefore, ultrasound energy is not uniformly distributed across tissues, and structures with different compositions may experience substantially different acoustic energy deposition despite being exposed to the same incident ultrasound field [34].

Adipose tissue has a relatively low acoustic attenuation compared with muscle, connective tissue and particularly bone. It may therefore permit a relatively greater proportion of incident ultrasound energy to propagate toward deeper structures. Nevertheless, subcutaneous adipose tissue should not be considered acoustically irrelevant. Its thickness, geometry and interfaces with neighbouring tissues can alter propagation and local energy distribution, meaning that the amount of energy reaching a deeper therapeutic target may differ substantially between individuals even when identical treatment parameters are applied [36].

Skeletal muscle demonstrates greater attenuation than adipose tissue and also exhibits acoustic anisotropy. Ultrasound propagation and attenuation vary according to the orientation of the acoustic beam relative to muscle fibres, with reported attenuation coefficients differing between propagation parallel and perpendicular to fibre direction. Consequently, muscle cannot be regarded as an acoustically homogeneous medium, and both muscle architecture and transducer orientation may influence the distribution of ultrasound energy within the target [34]. Experimental intramuscular thermometry has additionally demonstrated that the magnitude and rate of muscle heating depend strongly on ultrasound frequency and exposure parameters, with 3-MHz ultrasound producing more rapid superficial heating than 1-MHz ultrasound under comparable treatment conditions [37].

Collagen-rich connective tissues appear to interact more strongly with therapeutic ultrasound than tissues with lower attenuation coefficients. Tendons, ligaments and related connective structures contain a high proportion of organised collagen and exhibit relatively greater acoustic attenuation than adipose tissue and muscle. Human experimental studies have demonstrated substantial temperature elevations within superficial tendons during continuous therapeutic ultrasound. In the patellar tendon, for example, continuous 3-MHz ultrasound produced marked intratendinous temperature increases, with the magnitude of heating additionally influenced by the size of the treatment area [38].

Similar findings have been demonstrated in the Achilles tendon. During continuous 3-MHz therapeutic ultrasound, considerable increases in intratendinous temperature were recorded, although the magnitude of the response was strongly influenced by the coupling method used between the transducer and skin. These findings illustrate an important principle of energy–tissue interaction: a tissue may possess favourable acoustic characteristics for energy deposition, but the amount of energy ultimately reaching that tissue remains dependent on the efficiency of energy transmission from the applicator and through the intervening tissues [38].

Tissue interfaces further modify the distribution of ultrasound energy. When an acoustic wave encounters a boundary between tissues with different acoustic impedances, part of the energy is transmitted and part is reflected. Although this principle is particularly relevant at soft tissue–bone interfaces, interfaces between soft tissues should not be ignored. Experimental and computational investigations have demonstrated that skin–fat and fat–muscle boundaries can influence local temperature distribution during ultrasonic diathermy, indicating that the pathway between the transducer and therapeutic target should be regarded as a sequence of acoustically distinct tissues rather than as a homogeneous medium [36].

Bone represents an important example of tissue-dependent ultrasound interaction because its acoustic attenuation is markedly greater than that of many soft tissues. In addition, the substantial acoustic impedance mismatch between soft tissue and cortical bone can produce reflection of incident acoustic energy. Experimental investigations using bone phantoms and ex vivo human bone specimens have demonstrated substantial temperature elevations during therapeutic ultrasound exposure. The magnitude and spatial distribution of heating are influenced by exposure parameters and the physical characteristics of the tissues and interfaces encountered [36,39,40]. Consequently, high local energy interaction at bone should not automatically be interpreted as therapeutically advantageous, since excessive heating at the periosteal or soft tissue–bone interface may also represent a safety consideration.

The interaction occurring at the soft tissue–bone boundary is particularly relevant to structures such as entheses. At these locations, a collagen-rich tendon or ligament transitions into mineralised tissue, creating a highly heterogeneous acoustic environment. Reflection from cortical bone, absorption within adjacent tissues and interference between incident and reflected waves may modify the local distribution of acoustic energy. Experimental modelling has demonstrated that heating close to bone can result from a combination of direct energy absorption and reflected-wave phenomena in the adjacent soft tissues [41,42]. This provides a biophysical rationale for considering the enthesis as a distinct acoustic target rather than treating it as equivalent to either isolated tendon or muscle.

Depth must therefore be considered together with frequency and tissue composition. The conventional distinction between 1-MHz ultrasound for deeper targets and 3-MHz ultrasound for superficial targets reflects the greater attenuation of higher-frequency acoustic waves. However, target depth alone cannot predict the amount of energy available at a particular structure. Intensity, exposure duration, beam characteristics, tissue composition, perfusion, coupling conditions and the interfaces encountered during propagation collectively determine the resulting energy distribution and temperature response [34,38].

These principles indicate that therapeutic ultrasound should not be conceptualised simply as an energy source with a predetermined “penetration depth.” Rather, the body represents a heterogeneous acoustic pathway in which the energy reaching the target depends on the sequence and characteristics of the tissues encountered. A superficial tendon, a deep muscle beneath a substantial adipose layer and an enthesis adjacent to cortical bone therefore represent fundamentally different acoustic environments, even when identical ultrasound parameters are selected [34,36].

From an energy–tissue matching perspective, ultrasound may therefore demonstrate comparatively greater physical interaction with collagen-rich tissues and particularly with regions adjacent to bone because of differences in attenuation, absorption and acoustic impedance. Conversely, tissues characterised by lower attenuation may permit a greater proportion of the acoustic energy to continue propagating toward deeper structures. These observations provide a biophysical rationale for tissue-specific selection of ultrasound frequency and exposure parameters; however, stronger acoustic interaction or greater tissue heating should not be considered synonymous with greater therapeutic effectiveness. The clinical value of ultrasound for any particular disorder must ultimately be established independently through clinical evidence [34,40].

### 6.2. Photobiomodulation/LASER–Tissue Interaction

Photobiomodulation (PBM) delivers non-ionising optical energy, typically within the red and near-infrared (NIR) regions of the electromagnetic spectrum. Unlike therapeutic ultrasound, where energy is propagated as mechanical pressure waves, PBM depends on the propagation of photons through biological tissues and their subsequent interaction with endogenous absorbing molecules. Once light reaches the tissue surface, part of the incident energy is reflected, while the remainder enters the tissue and undergoes wavelength-dependent absorption and scattering. These processes progressively reduce the optical energy available with increasing tissue depth and determine both the spatial distribution of light and the optical dose potentially available to the therapeutic target [43].

Wavelength is one of the principal determinants of light–tissue interaction. Biological tissues contain numerous endogenous absorbers, including melanin, haemoglobin, water, lipids and other molecular constituents, each possessing wavelength-dependent absorption characteristics. Scattering is additionally influenced by tissue microstructure and differences in refractive index. Red and NIR wavelengths are commonly selected for PBM because the combined effects of absorption and scattering are relatively favourable for tissue transmission within this spectral region compared with shorter visible wavelengths. Nevertheless, the concept of an optical “therapeutic window” should not be interpreted as indicating unrestricted penetration; substantial attenuation still occurs within relatively superficial tissue layers [44].

Skin constitutes the first major optical barrier during transcutaneous PBM. At the skin surface, a proportion of incident photons is reflected, while photons entering the epidermis and dermis undergo repeated scattering and absorption. Melanin and haemoglobin are particularly important chromophores influencing this process, and differences in pigmentation may therefore modify the amount of optical energy transmitted toward deeper structures. A recent human in vivo investigation comparing 660- and 830-nm laser irradiation demonstrated significant effects of wavelength and melanin index on transmitted optical power, confirming that identical surface irradiation does not necessarily result in equivalent energy transmission between individuals [45].

The distinction between red and NIR wavelengths becomes particularly relevant when deeper musculoskeletal tissues are targeted. Red wavelengths generally undergo greater attenuation within superficial tissues, whereas NIR wavelengths can provide comparatively greater transmission through skin and underlying tissues. Experimental comparison of 905- and 1064-nm laser irradiation through ex vivo porcine skin and bovine muscle demonstrated consistently greater transmission for 1064 nm, particularly within the first 10 mm of tissue, although the absolute differences between the two wavelengths became progressively smaller with increasing tissue thickness. These findings demonstrate that increasing wavelength within the NIR spectrum may modify penetration, but wavelength alone cannot overcome the substantial attenuation produced by increasing tissue depth [46].

Adipose tissue represents an important intervening layer for PBM directed toward deeper musculoskeletal targets. Its optical behaviour differs from that of skin and muscle because absorption and scattering depend on tissue composition, lipid content, water content and microstructure. Experimental measurements using NIR therapeutic laser devices have demonstrated tissue-specific differences in transmission through fat, skin and muscle. Consequently, subcutaneous adipose thickness can influence the optical dose reaching an underlying muscle or tendon, and identical surface fluence should not be assumed to produce an identical target-tissue dose in individuals with different tissue geometries [47].

Skeletal muscle represents a common target for PBM, particularly in exercise and rehabilitation applications. However, the optical energy reaching muscle is determined first by transmission through the skin and subcutaneous tissues. Direct experimental measurements of therapeutic NIR laser devices demonstrated rapid attenuation as light propagated through muscle, with only a small fraction of incident optical power remaining after transmission through approximately 15 mm of muscle tissue. Thus, although NIR light can reach skeletal muscle, the energy available within deeper portions of a muscle may be substantially lower than the nominal energy delivered at the skin surface [47].

Tendon represents a particularly interesting target from an energy–tissue interaction perspective because many tendons are relatively superficial but may still be separated from the applicator by skin, subcutaneous tissue and, depending on the anatomical site, additional soft tissues. Human in vivo measurements of 660- and 830-nm light transmission through the calcaneal tendon region demonstrated measurable transmission but also substantial attenuation, with wavelength, pigmentation and device characteristics influencing transmitted power [45]. Earlier ex vivo human Achilles-tendon experiments similarly demonstrated that only a fraction of incident optical energy was transmitted through the complete tissue sample and that additional attenuation occurred within the tendon itself, reinforcing the importance of distinguishing surface irradiation from the dose actually interacting with the tendon [48].

The interaction of light with bone introduces further complexity. Bone is optically heterogeneous, consisting of mineralised matrix, collagen, water, blood-containing marrow and multiple structural interfaces capable of producing substantial scattering. Experimental measurements using therapeutic NIR laser devices demonstrated markedly lower transmission through bone-containing tissue than through muscle of equivalent overall thickness. In one experimental model, approximately 1.5% of initial optical power remained after transmission through 15 mm of muscle, whereas only approximately 0.25% was transmitted through tissue of similar thickness containing approximately 13 mm of bone. These observations indicate that bone constitutes a substantial optical barrier to transcutaneous light propagation [47].

Recent reviews of PBM for bone regeneration similarly emphasise that both absorption and scattering restrict the amount of external light reaching osseous targets. Although NIR wavelengths provide more favourable transmission than shorter wavelengths, cortical and trabecular bone remain highly scattering media, and the optical pathway to bone frequently includes skin, adipose tissue and muscle before the photons reach the intended osseous structure. Consequently, biological effects observed when bone cells or experimental bone defects are directly irradiated should not automatically be extrapolated to transcutaneous treatment of deeply located human bone [49].

The physical interaction of PBM differs fundamentally from modalities whose primary therapeutic stimulus is thermal or mechanical. For PBM, photons reaching the target must be absorbed by molecular photoacceptors capable of initiating photochemical or photophysical responses. Proposed mechanisms include interactions with mitochondrial chromophores and subsequent modulation of cellular signalling, although the identity and relative importance of specific photoacceptors remain areas of continuing investigation. Therefore, the relevant therapeutic quantity is not simply the energy emitted by the laser or LED but the optical energy that survives propagation and is subsequently absorbed by biologically relevant molecules within the target tissue [43,44].

This distinction has important implications for PBM dosimetry. Parameters such as wavelength, radiant power, irradiance, radiant exposure, treatment duration, beam area and application technique describe the energy delivered at or near the tissue surface, but they do not directly specify the dose received by a deeply located target. Recent PBM literature has therefore emphasised the importance of effective penetration depth and target-tissue dose estimation rather than reliance solely on surface radiant exposure. Without consideration of attenuation through intervening tissues, two treatments delivering an identical surface dose may expose their intended targets to substantially different quantities of optical energy [43].

PBM should therefore not be conceptualised as a modality possessing a single predetermined penetration depth. Optical energy decreases progressively with depth through wavelength-dependent absorption and scattering, and its distribution depends on skin pigmentation, tissue composition, anatomical location, adipose thickness and the nature and depth of the therapeutic target. A superficial Achilles tendon, a muscle located beneath several centimetres of soft tissue and a deeply situated bone therefore represent fundamentally different optical environments, even when the same wavelength and surface radiant exposure are applied [45].

From an energy–tissue matching perspective, these findings suggest that PBM possesses its strongest physical rationale when the selected wavelength can deliver sufficient optical energy through the intervening tissues to the intended target and when biologically relevant photon absorption can occur at that location. Superficial skin and connective-tissue targets therefore present fewer optical barriers than deeply located muscle, enthesis or bone. However, greater photon transmission should not be interpreted as equivalent to greater clinical effectiveness. The critical sequence is surface energy delivery → tissue transmission → target optical dose → molecular absorption → biological response, and failure at any stage may limit the intended therapeutic effect despite apparently appropriate device parameters [43].

### 6.3. Diathermy–Tissue Interaction

Diathermy applies high-frequency electromagnetic energy to biological tissues to produce thermal and, depending on exposure parameters, potentially non-thermal effects. In rehabilitation, shortwave diathermy (SWD) commonly operates at 27.12 MHz, whereas microwave diathermy (MWD) uses substantially higher frequencies. Unlike therapeutic ultrasound, diathermy does not rely on mechanical-wave propagation; electromagnetic fields interact with the electrical properties of biological tissues, producing energy deposition and conversion into heat [30].

The distribution of energy during SWD depends strongly on frequency, power, pulse characteristics, applicator configuration and treatment duration. Consequently, identical treatment times do not necessarily produce equivalent tissue heating. A systematic review of 11 studies involving 240 participants reported temperature increases ranging from approximately 0.3 °C with low-dose pulsed SWD to 7.9 °C with continuous capacitive SWD, demonstrating the strong dependence of tissue heating on application parameters [50].

The method of electromagnetic energy delivery also determines its distribution within tissues. Capacitive SWD primarily establishes an alternating electric field, whereas inductive applicators generate an alternating magnetic field that induces electrical currents within conductive tissues. Experimental tissue models demonstrate that different SWD applicators produce markedly different patterns of specific energy absorption and heating, with some configurations preferentially heating subcutaneous tissue and others producing greater energy deposition within muscle [51]. Human evidence similarly indicates that capacitive and inductive applications generate different superficial and intramuscular temperature responses [52].

Skin and superficial tissues may receive considerable thermal exposure during capacitive SWD. In a randomized crossover trial comparing three capacitive electrode arrangements, all configurations increased skin temperature, while the coplanar arrangement produced the greatest temperature increase, heated the largest surface area and demonstrated the slowest post-treatment temperature decay. These findings demonstrate that electrode geometry can substantially modify the spatial distribution of superficial heating even when the operating frequency and treatment duration remain unchanged [50].

Adipose tissue represents an important component of the electromagnetic pathway toward deeper therapeutic targets. Experimental tissue-substitute models demonstrate that energy deposition is not uniformly distributed between subcutaneous fat and underlying muscle and that their relative heating depends strongly on the SWD applicator. Some applicator configurations produced greater specific energy absorption and heating within the simulated adipose layer, whereas others preferentially heated the underlying muscle [51]. Adipose tissue should therefore not be regarded simply as a passive layer between the applicator and a deeper target.

Skeletal muscle can undergo substantial deep heating during appropriately configured SWD. Across experimental studies, intramuscular temperature responses varied considerably according to application technique, with inductive pulsed SWD producing increases approaching 4.6 °C in healthy muscle. This demonstrates that electromagnetic energy can generate clinically relevant heating within deeper muscle rather than limiting thermal effects to superficial tissues [52].

The relationship between frequency and target depth is particularly evident with microwave diathermy. In human muscle, comparison of 434- and 2450-MHz microwave systems demonstrated distinct heating distributions. Maximum heating with 434 MHz occurred at approximately 2 cm, whereas the maximum with 2450 MHz occurred at approximately 1 cm. Significant differences were also observed in peak muscle temperature, temperature increase and time to peak temperature, demonstrating that frequency and applicator characteristics strongly influence the depth and magnitude of electromagnetic energy deposition [31].

These observations challenge the concept that diathermy possesses a single predetermined “penetration depth.” The physical stimulus available at the target depends on the interaction between electromagnetic frequency, applicator type, field geometry, power, pulse characteristics and the tissues positioned within the field. Consequently, a deep muscle treated using an inductive SWD technique represents a substantially different energy–tissue environment from superficial tissues treated with capacitive SWD or muscle exposed to microwave energy at a different frequency [31,51].

Direct evidence concerning preferential energy deposition in tendon, ligament, enthesis and bone is considerably less developed than that available for skin and skeletal muscle. Although electromagnetic diathermy has been investigated clinically for several musculoskeletal conditions, improvement in a tendon, joint or periarticular disorder does not demonstrate preferential electromagnetic energy deposition within the pathological structure itself. Accordingly, strong tissue-specific claims for these structures should remain conservative in the absence of direct dosimetric or temperature measurements [53].

This distinction between physical interaction and clinical effectiveness is important. A recent systematic review and meta-analysis of electromagnetic diathermy for musculoskeletal disorders reported potential benefits for some clinical outcomes, but the certainty of evidence was predominantly low or very low and considerable methodological heterogeneity was present. Demonstrating that electromagnetic energy can produce meaningful tissue heating therefore establishes biophysical plausibility but does not independently demonstrate therapeutic efficacy for a particular pathology [53].

From an energy–tissue matching perspective, diathermy appears capable of producing substantially different thermal distributions according to the electromagnetic source and application technique. Capacitive SWD may favour greater superficial heating, whereas appropriate inductive SWD can generate substantial intramuscular heating; microwave frequency further modifies the depth at which maximal heating occurs. For tendon, ligament, enthesis and bone, however, current evidence does not justify equally strong claims of preferential target interaction. Diathermy should therefore be considered according to the desired depth and spatial distribution of electromagnetic energy rather than prescribed solely according to a diagnostic label [31,50].

### 6.4. TECAR/CRET–Tissue Interaction

Capacitive–resistive electric transfer (CRET), commonly referred to as TECAR therapy, applies radiofrequency electrical energy through an active electrode while a return electrode completes the electrical circuit through the body. Frequencies commonly used in rehabilitation are approximately 0.3–1.2 MHz, although many CRET devices operate around 448–600 kHz. Two principal application modes are used—capacitive and resistive—which differ according to electrode configuration and consequently modify the electrical and thermal interaction with tissues [54].

The distinction between capacitive and resistive application should not, however, be interpreted as absolute tissue selectivity. In capacitive mode, the active electrode is insulated, whereas the resistive electrode is uncoated, altering the electrical characteristics of the circuit. Experimental evidence demonstrates that both configurations can produce current flow and temperature changes in superficial and deeper tissues. Thus, the commonly proposed distinction of capacitive application for superficial or water-rich tissues and resistive application for deeper or denser tissues represents an oversimplification of a more complex interaction between electrode configuration, tissue impedance and applied power [32,55].

This complexity is demonstrated particularly well at the Achilles tendon and gastrocnemius musculotendinous junction. Direct cadaveric measurements showed that both capacitive and resistive protocols produced measurable temperature changes at superficial and deeper locations. High-power resistive application generated the greatest temperature increases within the Achilles tendon and musculotendinous junction, whereas lower-power protocols produced smaller thermal changes despite measurable current transmission. These findings demonstrate an important distinction between electrical energy transfer and subsequent thermal response: current reaching a target does not necessarily imply substantial tissue heating [32].

Similar findings have been reported around the knee. Measurements from superficial tissues, patellar tendon, intra-articular structures and muscle demonstrated that CRET current reached anatomical structures at different depths, whereas temperature responses varied substantially according to power and application mode. High-power protocols produced considerably greater heating than low-power protocols, despite electrical current remaining measurable during lower-power applications. Target exposure therefore appears to depend on the interaction between current pathway, tissue electrical characteristics and treatment parameters rather than anatomical depth alone [55].

Evidence from the shoulder further challenges rigid capacitive–resistive tissue classifications. Measurements at the skin, supraspinatus tendon and glenohumeral capsule demonstrated current flow and thermal responses at different anatomical depths. High-power resistive application produced the largest temperature increases, including within the supraspinatus tendon and joint capsule. These findings suggest that power and electrical configuration may be more important determinants of deep-tissue heating than the simple assignment of individual tissues to capacitive or resistive modes [55].

An important limitation is that much of the direct tissue-specific evidence derives from cadaveric experiments. These models permit invasive measurement of current and temperature within tendon, muscle and joint structures but do not reproduce physiological blood flow, thermoregulation or dynamic changes in tissue electrical properties. Their findings therefore provide valuable evidence of physical plausibility and energy distribution, but should not be interpreted as direct estimates of in-vivo tissue dose [32,55].

From an energy–tissue interaction perspective, TECAR/CRET should therefore not be reduced to the conventional concept of “capacitive for soft tissue” and “resistive for dense tissue.” Available experimental evidence instead suggests that electrode configuration, power, current pathway and tissue electrical properties collectively determine energy distribution. Importantly, current transmission, temperature elevation and clinical effectiveness represent distinct levels of evidence. TECAR prescription should therefore consider the intended electrical and thermal interaction at the target tissue, rather than relying solely on predetermined tissue labels assigned to capacitive or resistive modes [54].

### 6.5. Extracorporeal Shockwave Therapy–Tissue Interaction

Extracorporeal shockwave therapy (ESWT) delivers transient acoustic pressure waves that transfer mechanical energy into biological tissues. Focused shockwaves (fESWT) are generated electrohydraulically, electromagnetically or piezoelectrically and converge toward a defined focal region. In contrast, radial shockwave therapy generates diverging pressure waves with their highest energy close to the applicator. Radial waves lack several physical characteristics of true focused shockwaves and are therefore more accurately described as radial pressure waves. Consequently, focused and radial devices should not be regarded as physically interchangeable forms of energy delivery [56].

Once introduced into biological tissues, the propagation characteristics of acoustic pressure waves depend on the physical properties of the emitted waveform and the tissues encountered. For radial pressure wave therapy, experimental measurements and computational modelling have demonstrated depth-dependent changes in pressure amplitude and mechanical stress distribution. Differences in acoustic impedance between adjacent tissues may further influence wave transmission and reflection. These findings are specific to radial pressure wave propagation and should not be directly extrapolated to focused shockwaves, which exhibit different pressure profiles and spatial energy distributions [19].

This becomes particularly important near bone. Recent computational modelling specifically examining the soft-tissue–bone boundary demonstrated that reflection from bone alters radial pressure-wave propagation within adjacent soft tissue. Energy flux density at a target was influenced by both the initial pressure and the distance between the target and the reflecting bone surface. Bone therefore represents not merely a target or barrier but an acoustic boundary capable of modifying the local mechanical-energy distribution [57].

The biological consequences of extracorporeal acoustic stimulation are thought to involve mechanotransduction, through which mechanical forces are converted into cellular and molecular responses. Experimental investigations have reported changes involving cell proliferation, extracellular matrix regulation, angiogenic signalling and tissue remodelling following shockwave exposure. However, the magnitude and characteristics of these responses depend on the type of acoustic stimulus, exposure parameters and biological model investigated. Evidence obtained using focused shockwaves should therefore be distinguished from evidence involving radial pressure waves, rather than assuming equivalent biological responses at nominally similar device settings. Furthermore, demonstration of a cellular response does not establish that the same mechanism is responsible for clinical improvement [58].

Tendon represents an important anatomical target because its collagen-rich extracellular matrix is mechanically responsive. Experimental investigations using extracorporeal acoustic stimulation have reported changes in tenocyte activity, neovascularisation, extracellular matrix components, metalloproteinases, cytokines and lubricin. Nevertheless, these findings should be interpreted according to the specific device type and exposure characteristics employed in each investigation. Dose-dependent responses reported for focused shockwaves cannot automatically be generalized to radial pressure wave therapy, and vice versa. Excessive mechanical exposure may also produce adverse structural responses. Accordingly, tendon interaction should not be interpreted according to a simple “more energy is better” model; the delivered mechanical stimulus must remain within the biological tolerance of the target tissue [59].

Bone and tendon–bone regions provide particularly relevant examples of device-dependent acoustic interaction. Focused shockwaves can concentrate acoustic energy within a defined focal region, whereas radial pressure waves exhibit their greatest pressure near the applicator, with pressure generally decreasing as propagation distance increases. Consequently, the mechanical exposure of a skeletal target differs between focused and radial devices, even when treatments are described using superficially similar parameters. Bone-related ESWT studies employ heterogeneous exposure protocols, including different pressure characteristics, energy flux densities, pulse numbers and targeting strategies. Reported associations between treatment parameters and clinical outcomes should therefore be interpreted within the specific device category investigated. Findings derived from focused shockwave protocols should not be pooled conceptually with radial pressure wave evidence when discussing penetration, mechanical dose or biological effectiveness [60].

From an energy–tissue interaction perspective, extracorporeal acoustic therapies produce predominantly mechanical stimuli, but focused shockwaves and radial pressure waves differ substantially in their physical characteristics and spatial exposure distributions. Effective target interaction depends on the specific waveform, pressure characteristics, anatomical depth, tissue composition and acoustic interfaces encountered. These factors must be evaluated separately for focused and radial devices. Although mechanical stimulation may initiate mechanotransduction, physical interaction, biological response and clinical effectiveness remain distinct levels of evidence. Consequently, device selection and treatment parameters should be matched to the intended anatomical target and the physical stimulus required, rather than treating focused and radial applications as interchangeable interventions [61].

### 6.6. Pulsed Electromagnetic Field–Tissue Interaction

Pulsed electromagnetic field (PEMF) therapy exposes biological tissues to time-varying electromagnetic fields generated by coils or related applicators. Unlike ultrasound, ESWT or photobiomodulation, PEMF does not require direct transfer of mechanical waves or photons into the target. A changing magnetic field can penetrate biological tissues and induce secondary electric fields and currents according to electromagnetic induction. Consequently, PEMF interaction is better conceptualised as field exposure and induced electrical stimulation rather than conventional energy absorption at a discrete tissue target [62].

The resulting biological exposure depends strongly on the physical characteristics of the applied field. Magnetic flux density, frequency, pulse waveform, pulse duration, repetition frequency, exposure duration and applicator geometry vary considerably between PEMF systems. This heterogeneity is important because different parameter combinations can generate substantially different induced electrical environments within tissues. Therefore, describing an intervention simply as “PEMF therapy” provides insufficient information to define the physical stimulus received by the target tissue [27].

A distinctive feature of PEMF is that tissue depth does not restrict exposure in the same manner as optical attenuation in PBM or pressure-wave attenuation in radial ESWT. Magnetic fields can traverse skin and intervening soft tissues without requiring preferential absorption within these layers. Nevertheless, field penetration should not be equated with equivalent biological stimulation at every depth: the induced electric field depends on field strength and spatial distribution, tissue electrical characteristics and anatomical geometry. Thus, magnetic-field penetration and effective target interaction represent different concepts [62].

Bone is the musculoskeletal tissue for which PEMF has received the greatest mechanistic attention. Experimental evidence indicates that PEMF exposure can influence osteoblast activity, osteogenic differentiation and signalling pathways involved in bone formation and remodelling. Proposed mechanisms include membrane-level signalling involving adenosine receptors and downstream pathways such as Wnt/β-catenin and MAPK. This provides biological plausibility for bone as a PEMF target, although the precise response remains dependent on the electromagnetic exposure parameters [63].

PEMF effects have also been investigated in cartilage, muscle, tendon and other connective tissues, but the evidence is less consistent and treatment parameters vary markedly between studies. This variability makes it difficult to establish a simple hierarchy in which one tissue can be considered intrinsically more receptive to PEMF than another. Unlike modalities characterised by obvious preferential attenuation or absorption, PEMF tissue matching may therefore depend more strongly on cellular responsiveness to the induced electrical stimulus and exposure parameters than on preferential physical deposition of energy within a particular tissue [64].

Finally, biological plausibility should again be separated from clinical effectiveness. A 2024 systematic review of randomized controlled trials found no significant improvement in acute fracture healing with PEMF, despite substantial experimental evidence supporting effects on bone cells and osteogenic pathways. From an energy–tissue interaction perspective, PEMF therefore illustrates particularly clearly that physical access to a target and biological responsiveness do not guarantee clinical benefit. Prescription should consider field parameters, induced electrical stimulation and target biology rather than if magnetic-field penetration alone constitutes an adequate therapeutic dose [65].

## 7. Tissue–Energy Interaction Framework

The preceding evidence demonstrates that electrophysical agents cannot be considered interchangeable sources of therapeutic energy. Acoustic waves, photons, radiofrequency currents, shockwaves and electromagnetic fields interact with biological tissues through fundamentally different physical processes. Moreover, the magnitude and spatial distribution of these interactions depend simultaneously on the characteristics of the energy source and those of the tissues encountered. Consequently, consideration of the intended anatomical target and the physical interaction required should form part of modality selection, alongside the available evidence of clinical effectiveness, rather than relying exclusively on diagnostic labels or standardized protocols [22,66].

**Figure 1:**
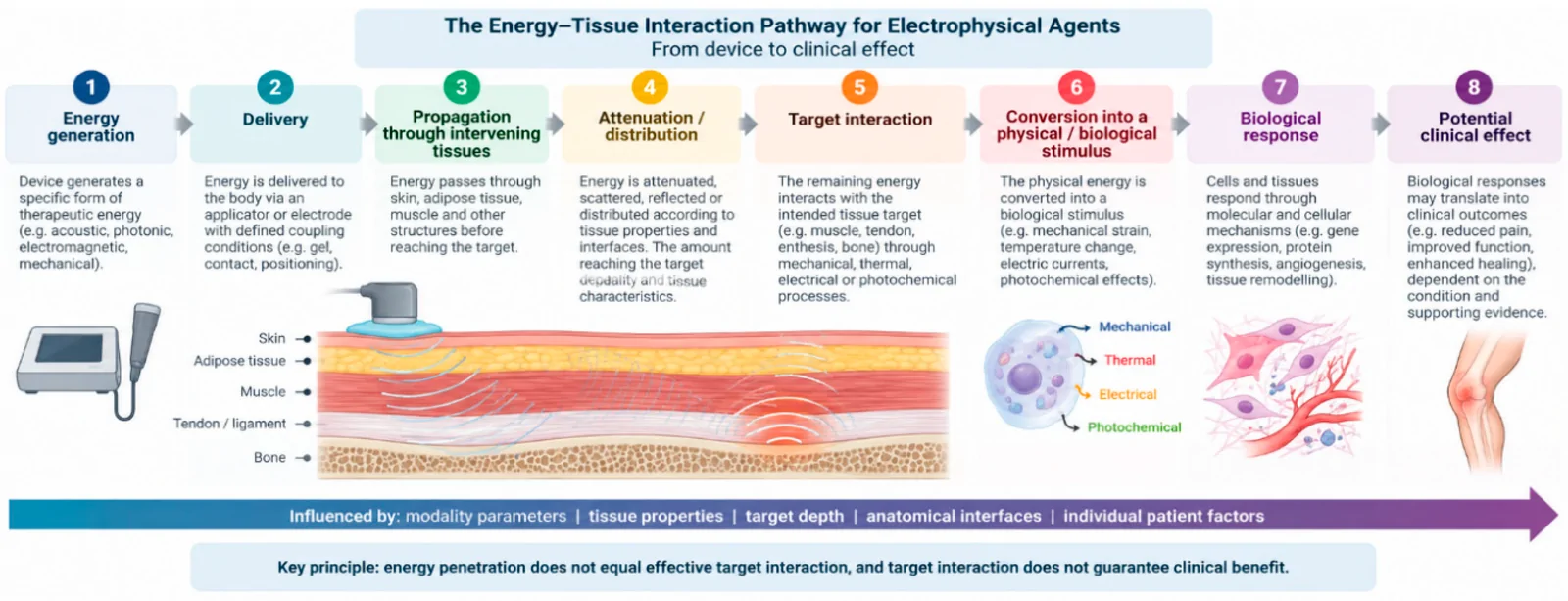
Tissue–Energy Interaction Framework.

The proposed Tissue Energy Interaction Framework (TEIF) conceptualises EPA treatment as a sequential pathway (Figure 1).

Each stage can modify the eventual therapeutic stimulus. A modality may generate sufficient energy at the applicator yet deliver only a small fraction to a deep target because of attenuation, scattering or reflection. Conversely, energy may reach a target but produce limited biological interaction if the delivered physical stimulus is insufficient or the tissue does not respond meaningfully to that stimulus. Therefore, energy penetration should be distinguished from effective energy–tissue interaction at the target [43,67].

This distinction becomes apparent when comparing modalities. Ultrasound is influenced by acoustic attenuation, absorption and reflection, particularly at heterogeneous tissue interfaces. PBM is governed by wavelength-dependent optical absorption and scattering, making tissue composition and target depth important determinants of photon availability. Diathermy and TECAR distribute electromagnetic or electrical energy according to field configuration, current pathways and tissue electrical properties, producing parameter-dependent thermal responses. Focused shockwaves and radial pressure waves differ in their pressure profiles and spatial distributions of mechanical exposure and should therefore be considered separately. PEMF differs further because magnetic fields may traverse intervening tissues relatively readily, while the induced electrical environment depends on field parameters, tissue electrical characteristics and anatomical geometry [26].

Accordingly, the concept of penetration depth alone is insufficient for evaluating EPA–tissue interactions. Physical exposure at a target depends on the characteristics of the delivered energy, the tissues traversed, anatomical interfaces and treatment parameters. Furthermore, energy transmission, absorption, thermal responses, mechanical stimulation and biological responsiveness represent distinct phenomena that cannot be directly compared using a common scale of tissue interaction. Identical device settings may therefore produce different physical exposure distributions across anatomical regions or patients [28,67].

The Tissue–Modality Interaction Matrix (Table 2) summarizes these relationships through descriptive statements rather than ordinal classifications of tissue responsiveness. The matrix distinguishes established physical mechanisms from experimentally observed responses and acknowledges limitations arising from heterogeneous experimental models. In particular, tissue-specific TECAR findings derived from cadaveric investigations require cautious extrapolation to living tissues, while focused shockwave and radial pressure wave evidence must be interpreted according to the device type investigated. The matrix is intended to illustrate physical and biological plausibility rather than provide a validated ranking of modalities or predict clinical effectiveness.

Importantly, favourable energy–tissue matching establishes physical plausibility, not clinical efficacy. Successful interaction at the target is only an early step in the pathway toward potential therapeutic benefit. The resulting physical stimulus must initiate an appropriate biological response, and that response must ultimately translate into a clinically meaningful effect. Physical plausibility, biological plausibility and clinical effectiveness should therefore be regarded as distinct but complementary levels of evaluation, with evidence required at each level rather than assuming that demonstration of one establishes the others [68,69].

The TEIF consequently proposes a conceptual approach to integrating target-specific biophysical considerations into EPA selection. Clinicians may consider the intended tissue target, its anatomical depth and relevant physical characteristics; examine whether the proposed energy source and treatment parameters can plausibly produce the intended physical interaction; and evaluate whether biological and clinical evidence supports the intervention. However, TEIF is not a validated clinical decision-making algorithm and should not be used independently to determine treatment selection or effectiveness. Instead, it provides a biophysical perspective that may complement evidence-based practice and guide future experimental investigation and clinical validation [32,66].

## 8. Conclusions

Electrophysical agents remain widely used in rehabilitation, yet their prescription is frequently organised around diagnostic labels, established protocols or device-specific parameters rather than the physical interaction between the delivered energy and the intended biological target. This review proposes that a more fundamental question should precede modality selection: can the energy generated by a particular electrophysical agent reach and interact meaningfully with the intended target tissue?

The evidence reviewed demonstrates that this interaction differs substantially among modalities. Ultrasound is governed by acoustic transmission, attenuation, absorption and reflection; photobiomodulation by wavelength-dependent optical absorption and scattering; diathermy and TECAR by electromagnetic or electrical energy distribution and tissue electrical properties; ESWT by pressure-wave propagation, mechanical stress and interface effects; and PEMF primarily by magnetic-field exposure and induced electrical stimulation. Consequently, the same anatomical depth or diagnostic condition does not represent an equivalent physical environment for different forms of therapeutic energy.

The proposed Tissue Energy Interaction Framework (TEIF) integrates these principles through the sequence of energy generation, delivery, propagation, attenuation/distribution, target interaction, conversion into a physical or biological stimulus, biological response and potential clinical effect. This framework emphasises that energy penetration is not synonymous with effective target interaction and, equally importantly, that measurable target interaction does not guarantee therapeutic benefit.

EPA selection should therefore progress from identifying the intended tissue target and its relevant physical characteristics to determining which form of energy can plausibly reach and interact with that target, followed by selection of appropriate treatment parameters. Only thereafter should biological mechanisms and evidence of clinical effectiveness determine whether the intervention is justified. In this way, EPA prescription can be considered according to the hierarchy of physical plausibility → biological plausibility → clinical effectiveness.

The TEIF is intended as a conceptual framework rather than a replacement for evidence-based clinical decision-making. Its value lies in providing a biophysical foundation through which existing evidence, treatment parameters and future research can be interpreted. Future experimental and clinical studies should therefore report not only device settings and clinical outcomes but also consider the physical dose reaching the intended tissue target. Such an approach may help move electrophysical-agent prescription away from standardised protocol-driven application toward more rational, tissue-specific and mechanistically informed rehabilitation practice.

## 9. Recommendations for Future Research

Future research on electrophysical agents should move beyond reporting device settings and clinical outcomes alone and investigate the complete pathway between energy delivery, target-tissue exposure, biological response and clinical effect. A major priority should be the direct quantification of the physical stimulus reaching specific anatomical targets under clinically relevant treatment conditions. Depending on the modality, this may include acoustic pressure or energy deposition, photon fluence, tissue temperature, electrical current density, induced electric fields or mechanical stress.

Studies should systematically examine how target depth, tissue composition and intervening anatomical structures modify energy delivery. Attention should be given to tissue interfaces, such as fat–muscle, tendon–bone and soft tissue–bone boundaries, because these may substantially alter transmission, reflection, attenuation or energy distribution. Whenever possible, experimental measurements should progress from computational and phantom models to cadaveric models and ultimately to in-vivo human investigations.

A further priority is the development of tissue-specific dosimetry. Treatment parameters are commonly reported at the device or applicator level, although the nominal output may differ substantially from the physical dose reaching the intended target. Future studies should therefore distinguish between *device output*, *delivered energy* and *target-tissue dose*. This distinction may help explain why apparently similar protocols produce different biological or clinical outcomes.

Research should also investigate whether individual anatomical characteristics—including subcutaneous tissue thickness, target depth, tissue orientation and composition—require modification of treatment parameters. Such studies could determine whether patient-specific EPA prescription provides more reproducible target exposure than conventional fixed protocols and could ultimately support personalised dosimetry.

Importantly, mechanistic and clinical research should be more closely integrated. Studies demonstrating energy penetration or target interaction should subsequently determine whether the measured physical stimulus produces a relevant biological response and whether this response predicts meaningful clinical outcomes. This would permit direct testing of the proposed hierarchy of physical plausibility → biological plausibility → clinical effectiveness.

Finally, the proposed TEIF requires prospective validation. Future studies could compare conventional diagnosis-based EPA prescription with target-centred, biophysically informed selection based on tissue characteristics and predicted energy interaction. Validation of such an approach would determine whether incorporating energy–tissue principles improve treatment parameter selection, reproducibility and, ultimately, clinical outcomes.

## Figures and Tables

**Table 1:** Records for each modality search.

Modality	PubMed	Scopus	Total
**US**	246	1298	1544
**PBM**	359	1160	1519
**Diathermy**	28	51	79
**TECAR**	188	97	285
**ESWT**	157	230	387
**PEMF**	51	130	181
**Total**	**1029**	**2966**	**3995**

**Table 2:** Tissue–Modality Interaction.

Modality	Skin/Subcutaneous Tissue	Adipose Tissue	Muscle	Tendon/Ligament	Enthesis	Bone
**Therapeutic ultrasound**	Superficial heating depends on frequency and exposure parameters.	Acoustic propagation and attenuation; interfaces may modify distribution.	Frequency- and orientation-dependent attenuation; intramuscular heating can occur.	Collagen-associated attenuation; tendon heating has been measured.	Reflection and altered local field near the tendon–bone interface.	Strong attenuation and impedance mismatch; localized heating demonstrated experimentally.
**PBM/LASER**	Wavelength-dependent absorption and scattering by chromophores.	Scattering and absorption reduce photon availability.	Photon delivery decreases with depth.	Limited photon delivery; biological responses require target exposure.	Limited evidence for adequate target photon dose.	Optical access limited by intervening tissues; biological effects not established.
**Diathermy**	Superficial electromagnetic heating depends on applicator configuration and parameters.	Dielectric properties influence energy distribution and heating.	Intramuscular heating can occur under suitable configurations.	Local heating is physically possible, but direct tissue-specific evidence is limited.	Exposure depends on field distribution and surrounding tissues.	Preferential bone heating is not established.
**TECAR/CRET**	Current transmission and temperature changes depend on electrode mode and parameters.	Impedance and tissue geometry influence current pathways.	Parameter-dependent heating measured in cadaveric models.	Tendon heating demonstrated mainly in cadaveric studies.	Limited direct evidence for specific interaction.	Selective bone heating is not established.
**Focused ESWT**	Acoustic propagation through superficial tissues toward focus.	Propagation through intervening tissues; focal dose depends on tissue pathway.	Local mechanical exposure depends on focal position.	Mechanical stimulation may initiate biological responses.	Focal mechanical interaction at the tendon–bone region.	Targeted mechanical exposure at a defined focal region.
**Radial pressure waves**	Highest pressure generally occurs near the applicator.	Depth-dependent pressure attenuation.	Depth-dependent pressure and mechanical stress distribution.	Mechanical exposure depends on target depth and parameters.	Bone reflection can modify local pressure distribution.	Bone interfaces alter pressure-wave propagation.
**PEMF**	Magnetic-field exposure and induced electrical fields.	Magnetic field penetrates tissue; induced field varies with conductivity.	Induced electrical fields depend on tissue conductivity and parameters.	Biological responses have been investigated.	Limited direct evidence for specific interaction.	Osteogenic responses have been investigated; clinical effectiveness not established consistently.
**Predominant energy–tissue interaction (summary)**	↑ Energy transmission, attenuation and absorption (thermal/mechanical/photobiological)	↑ Energy absorption and scattering	↑ Energy absorption and mechanical/photobiological effects	↑ Energy absorption, current flow and mechanical/biological interaction	↑ Wave propagation and target interaction at tissue interfaces	↑ Energy interaction depends on tissue properties and interfaces

**■ Higher/well-described interaction, ■ Moderate/parameter-dependent, ■ Limited or uncertain evidence.** Note: The table summarizes physical energy–tissue interactions based on available experimental and modelling evidence. It does not represent a quantitative ranking or clinical effectiveness. Color categories are qualitative visual aids, not validated evidence grades. Cadaveric findings require cautious extrapolation to living tissues; focused shockwaves and radial pressure waves are considered separately.

## Data Availability

No new data were created or analyzed in this study. Data sharing is not applicable to this article.

## Supplementary Materials

The following supporting information can be downloaded at: https://www.mdpi.com/article/10.3390/jcm15218216/s1, Table S1: Database-specific literature search strategies.

## Abbreviations

The following abbreviations are used in this manuscript:

EPA	Electrophysical Agents
TECAR/CRET	Capacitive-resistive electric transfer
ESWT	Extracorporeal Shockwave Therapy
PEMF	Pulsed electromagnetic fields
TEIF	Tissue Energy Interaction Framework
PBM	Photobiomodulation
SWD	Shortwave Diathermy
MWD	Microwave Diathermy
NIR	Near Infrared
MAPK	Mitogen-activated protein kinase
