# Supplementary material for: Energy–Tissue Interaction in Physiotherapy: A Proposed Conceptual Framework for Electrophysical Agents
Source: J Clin Med. 2026 Oct 22;15(21):8216. doi: 10.3390/jcm15218216 (PMC13764133; doi:10.3390/jcm15218216)
Supplement: Supplementary file 1 [file jcm-15-08216-s001.zip › Supplementary Table S1.pdf]

Supplementary Table S1. Database-specific literature search strategies

Structured narrative review of tissue–energy interactions of electrophysical agents. Working supplement for verification against original PubMed and Scopus search histories.

Audit and reproducibility notes

• Eligibility window: publications dated 1 January 2016–1 September 2026.

• Recorded search totals: PubMed 1,029; Scopus 2,966; combined 3,995; after deduplication 3,271.

Table S1. Full database-specific queries

For each PubMed query, append: AND ("2016/01/01"[Date - Publication] : "2026/09/01"[Date - Publication]). For Scopus, append: AND PUBYEAR > 2015 AND PUBYEAR < 2027; are displayed separately so the original query bodies remain auditable.

Therapeutic ultrasound

PubMed | Recorded count: 246 |

```
("therapeutic ultrasound"[Title/Abstract] OR "ultrasonic diathermy"[Title/Abstract]) AND
("attenuation"[Title/Abstract] OR "absorption"[Title/Abstract] OR "tissue
heating"[Title/Abstract] OR "acoustic impedance"[Title/Abstract] OR "energy
deposition"[Title/Abstract]) AND ("muscle"[Title/Abstract] OR "tendon"[Title/Abstract] OR
"bone"[Title/Abstract] OR "adipose"[Title/Abstract] OR "tissue"[Title/Abstract])
```

Scopus | Recorded count: 1298

```
TITLE-ABS-KEY(("therapeutic ultrasound" OR "ultrasound therapy" OR "ultrasonic diathermy") AND
(attenuation OR absorption OR "tissue interaction" OR penetration OR temperature OR heating OR
"acoustic pressure" OR "acoustic radiation force"))
```

Photobiomodulation (PBM)

PubMed | Recorded count: 359

```
("Low-Level Light Therapy"[MeSH Terms] OR "photobiomodulation"[Title/Abstract] OR "low-level
laser therapy"[Title/Abstract] OR "low level light therapy"[Title/Abstract] OR "laser
phototherapy"[Title/Abstract]) AND ("absorption"[Title/Abstract] OR "scattering"[Title/Abstract]
OR "penetration"[Title/Abstract] OR "attenuation"[Title/Abstract] OR "optical
properties"[Title/Abstract] OR "light distribution"[Title/Abstract] OR "energy
deposition"[Title/Abstract] OR "dosimetry"[Title/Abstract] OR "tissue
interaction"[Title/Abstract])
```

Scopus | Recorded count: 1160

```
TITLE-ABS-KEY(("photobiomodulation" OR "low-level laser therapy" OR "low level light therapy" OR
"laser phototherapy") AND (absorption OR scattering OR penetration OR attenuation OR "optical
properties" OR "light distribution" OR "energy deposition" OR dosimetry OR "tissue interaction"))
```

Diathermy

PubMed | Recorded count: 28 |

```
("Diathermy"[MeSH Terms] OR "shortwave diathermy"[Title/Abstract] OR "short-wave
diathermy"[Title/Abstract] OR "microwave diathermy"[Title/Abstract] OR "pulsed
shortwave"[Title/Abstract] OR "pulsed short-wave"[Title/Abstract]) AND ("tissue
heating"[Title/Abstract] OR "temperature"[Title/Abstract] OR "energy absorption"[Title/Abstract]
OR "energy deposition"[Title/Abstract] OR "penetration"[Title/Abstract] OR "electromagnetic
field"[Title/Abstract] OR "specific absorption rate"[Title/Abstract] OR "thermal
distribution"[Title/Abstract] OR "tissue interaction"[Title/Abstract])
```

Scopus | Recorded count: 51 |

```
TITLE-ABS-KEY(("shortwave diathermy" OR "short-wave diathermy" OR "microwave diathermy" OR
"pulsed shortwave" OR "pulsed short-wave") AND ("tissue heating" OR temperature OR "energy
absorption" OR "energy deposition" OR penetration OR "electromagnetic field" OR "specific
absorption rate" OR "thermal distribution"))
```

TECAR / CRET

PubMed | Recorded count: 188 | Status: Reconstructed

```
("TECAR"[Title/Abstract] OR "capacitive resistive electric transfer"[Title/Abstract] OR
"capacitive-resistive electric transfer"[Title/Abstract] OR "capacitive resistive
therapy"[Title/Abstract] OR "capacitive resistive radiofrequency"[Title/Abstract] OR "448
kHz"[Title/Abstract]) AND ("temperature"[Title/Abstract] OR "heating"[Title/Abstract] OR "thermal
response"[Title/Abstract] OR "blood flow"[Title/Abstract] OR "tissue"[Title/Abstract] OR
"impedance"[Title/Abstract] OR "current distribution"[Title/Abstract])
```

Scopus | Recorded count: 97 | Status: Reconstructed

```
TITLE-ABS-KEY(("TECAR" OR "capacitive resistive electric transfer" OR "capacitive-resistive
electric transfer" OR "capacitive resistive therapy" OR "capacitive resistive radiofrequency")
AND (temperature OR heating OR "thermal response" OR "blood flow" OR tissue OR impedance OR
"current distribution"))
```

ESWT

PubMed | Recorded count: 157 | Status: Reconstructed

```
("Extracorporeal Shockwave Therapy"[MeSH Terms] OR "extracorporeal shock wave
therapy"[Title/Abstract] OR "extracorporeal shockwave therapy"[Title/Abstract] OR "focused
shockwave"[Title/Abstract] OR "radial shockwave"[Title/Abstract] OR "radial pressure
wave"[Title/Abstract]) AND ("penetration"[Title/Abstract] OR "attenuation"[Title/Abstract] OR
"pressure distribution"[Title/Abstract] OR "mechanical stimulation"[Title/Abstract] OR "energy
flux density"[Title/Abstract] OR "tissue interaction"[Title/Abstract] OR "bone
interface"[Title/Abstract] OR "mechanotransduction"[Title/Abstract])
```

Scopus | Recorded count: 230 | Status: Reconstructed

```
TITLE-ABS-KEY(("extracorporeal shock wave therapy" OR "extracorporeal shockwave therapy" OR
"focused shockwave" OR "radial shockwave" OR "radial pressure wave") AND (penetration OR
attenuation OR "pressure distribution" OR "mechanical stimulation" OR "energy flux density" OR
"tissue interaction" OR "bone interface" OR mechanotransduction))
```

PEMF

PubMed | Recorded count: 51 | Status: Reconstructed

```
("Electromagnetic Fields"[MeSH Terms] OR "pulsed electromagnetic field"[Title/Abstract] OR
"pulsed electromagnetic fields"[Title/Abstract] OR "PEMF"[Title/Abstract] OR "pulsed magnetic
field"[Title/Abstract]) AND ("tissue interaction"[Title/Abstract] OR "induced electric
field"[Title/Abstract] OR "conductivity"[Title/Abstract] OR "penetration"[Title/Abstract] OR
"bone healing"[Title/Abstract] OR "osteogenesis"[Title/Abstract] OR "cellular
response"[Title/Abstract] OR "electromagnetic stimulation"[Title/Abstract])
```

Scopus | Recorded count: 130 | Status: Reconstructed

```
TITLE-ABS-KEY(("pulsed electromagnetic field" OR "pulsed electromagnetic fields" OR PEMF OR
"pulsed magnetic field") AND ("tissue interaction" OR "induced electric field" OR conductivity OR
penetration OR "bone healing" OR osteogenesis OR "cellular response" OR "electromagnetic
stimulation"))
```

Summary of recorded counts

Modality	PubMed	Scopus	Combined
**Therapeutic ultrasound**	246	1298	1544
**Photobiomodulation (PBM)**	359	1160	1519
**Diathermy**	28	51	79
**TECAR / CRET**	188	97	285
**ESWT**	157	230	387
**PEMF**	51	130	181
**TOTAL**	1,029	2,966	3,995
